# Supplementary material for: A bacterial CARD–NLR-like immune system controls the release of gene transfer agents
Source: Nat Microbiol. 2026 Apr 16;11(6):1511–30. doi: 10.1038/s41564-026-02316-4 (PMC13236601; doi:10.1038/s41564-026-02316-4)
Supplement: Supplementary file 1 — Supplementary Figs. 1 and 2. [file 41564_2026_2316_MOESM1_ESM.pdf]

nature microbiology

Supplementary information https://doi.org/10.1038/s41564-026-02316-4

A bacterial CARD–NLR-like immune system controls the release of gene transfer agents

In the format provided by the authors and unedited

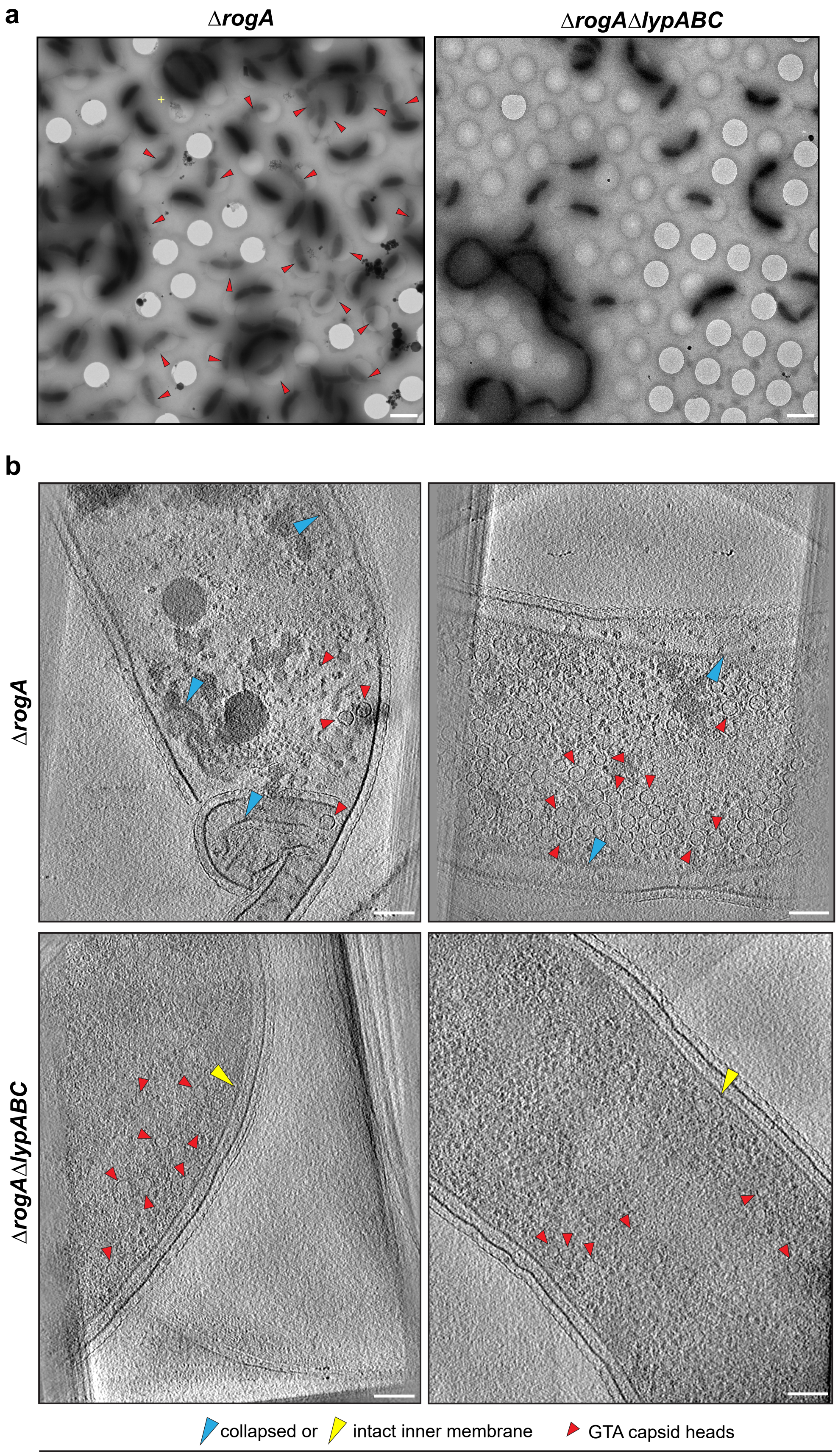

Supplementary Fig. 1. Cryo-electron microscopy and tomography of GTA-producing strains.
a. Cryo-electron microscopy search images from either the Δ*rogA* strain (left) or Δ*rogA*Δ*lypABC* strain (right). Red arrows indicate lysing ghost cells in the Δ*rogA* strain only. Scale bar: 2 µm. Images are representative of two independent repeats. **b.** Additional cryo-electron tomograms of Δ*rogA* (top row) and Δ*rogA*Δ*lypABC* (bottom row) cells to supplement **Fig. 3c**. Red arrows: GTA capsid heads, blue arrow: collapsed inner membrane, yellow arrow: intact inner membrane. Scale bar: 100 nm. Images are representative of two independent experiments.

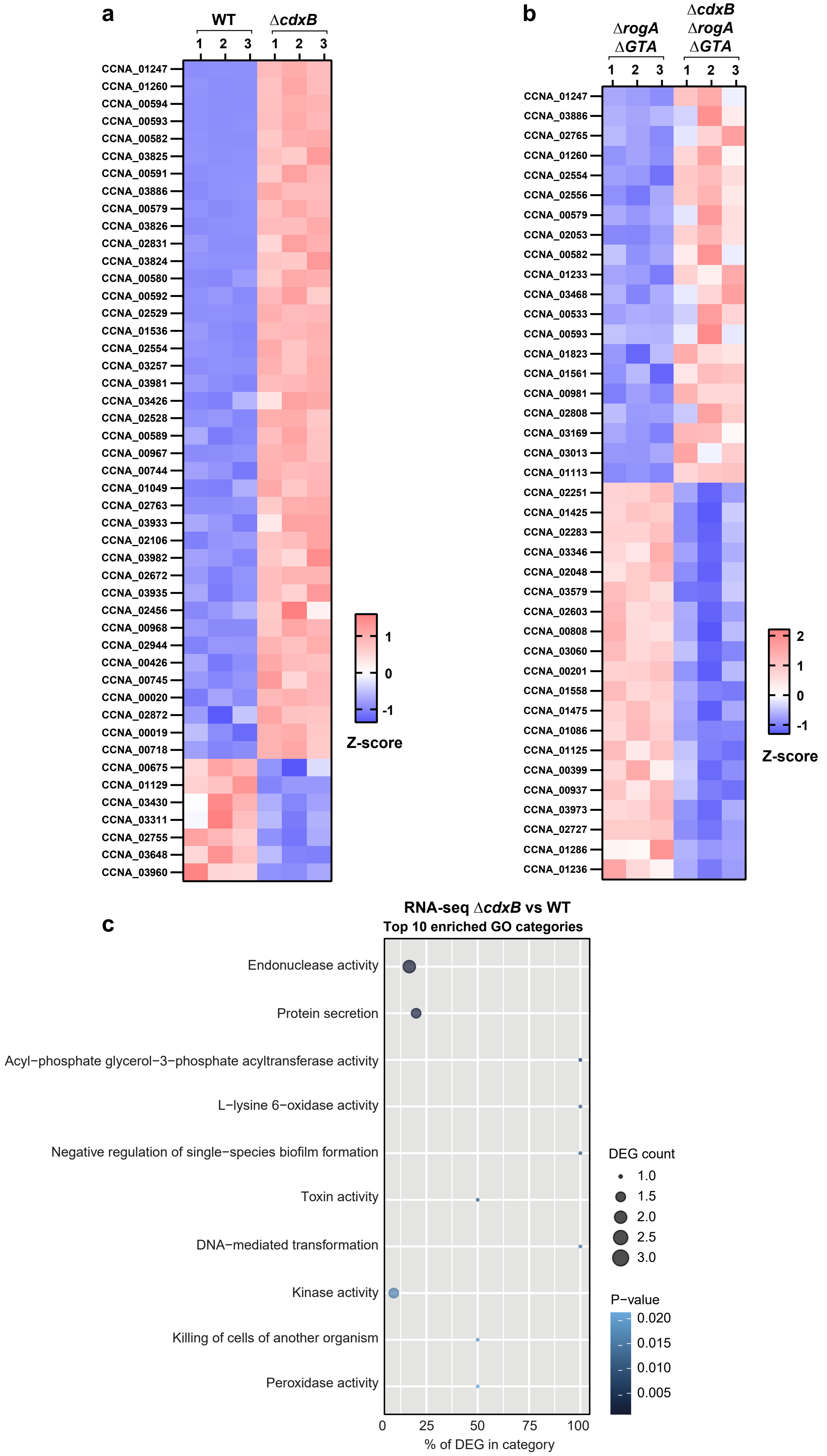

Supplementary Fig. 2. RNA-seq experiment heatmaps and gene ontology enrichment analyses.
Heatmaps showing the top differentially expressed genes for the RNA-seq experiments presented in **Fig. 6d (a)** and **Extended Data Fig. 10c (b)**. Normalised read counts for each of three replicates (1-3) were used to generate Z-scores to show the differential expression for each gene across replicates in each mutant background. Red or blue values indicate higher or lower expression, respectively. **c.** Top ten gene ontology (GO) categories enriched in RNA-seq data comparing Δ*cdxB* to wildtype. DESeq2 was used for differential expression analysis with default settings (Wald test followed by P-value adjustment for multiple comparisons using the Benjamini-Hochberg method). Data are presented as bubble plots in which the size of the bubble indicates the number of differentially expressed genes (DEG) within each GO category, and the bubble colour indicates the P-value (dark blue: more significant; light blue: less significant).
