# Supplementary material for: A bacterial CARD–NLR-like immune system controls the release of gene transfer agents
Source: Nat Microbiol. 2026 Apr 16;11(6):1511–30. doi: 10.1038/s41564-026-02316-4 (PMC13236601; doi:10.1038/s41564-026-02316-4)
Supplement: Supplementary file 2 — Reporting Summary [file 41564_2026_2316_MOESM2_ESM.pdf]

Corresponding author(s): Tung Le & Emma Banks
Last updated by author(s): 27.2.26

Reporting Summary

Nature Portfolio wishes to improve the reproducibility of the work that we publish. This form provides structure for consistency and transparency in reporting. For further information on Nature Portfolio policies, see our Editorial Policies and the Editorial Policy Checklist.

Statistics

For all statistical analyses, confirm that the following items are present in the figure legend, table legend, main text, or Methods section.

n/a	Confirmed	
☐	☒	The exact sample size (*n*) for each experimental group/condition, given as a discrete number and unit of measurement
☐	☒	A statement on whether measurements were taken from distinct samples or whether the same sample was measured repeatedly
☐	☒	The statistical test(s) used AND whether they are one- or two-sided<br>*Only common tests should be described solely by name; describe more complex techniques in the Methods section.*
☒	☐	A description of all covariates tested
☐	☒	A description of any assumptions or corrections, such as tests of normality and adjustment for multiple comparisons
☐	☒	A full description of the statistical parameters including central tendency (e.g. means) or other basic estimates (e.g. regression coefficient) AND variation (e.g. standard deviation) or associated estimates of uncertainty (e.g. confidence intervals)
☐	☒	For null hypothesis testing, the test statistic (e.g. *F*, *t*, *r*) with confidence intervals, effect sizes, degrees of freedom and *P* value noted<br>*Give P values as exact values whenever suitable.*
☒	☐	For Bayesian analysis, information on the choice of priors and Markov chain Monte Carlo settings
☒	☐	For hierarchical and complex designs, identification of the appropriate level for tests and full reporting of outcomes
☒	☐	Estimates of effect sizes (e.g. Cohen's *d*, Pearson's *r*), indicating how they were calculated

Our web collection on statistics for biologists contains articles on many of the points above.

Software and code

Policy information about availability of computer code

Data collection	Zeiss ZenBlue Version 2.3 microscope software, Amersham Imager 600 (GE Healthcare) western blot software
Data analysis	Adobe Illustrator v. 28 (Adobe), Excel 365 (Microsoft), Galaxy EU server, PyMOL v. 2.5.2, GraphPad Prism v. 10, UCSF Chimera X v. 1.9, ImageJ v. 1.53t, MicrobeJ v.13I, AlphaFold3 (https://alphafoldserver.com/). Custom code used to analyse Tn-seq and RNA-seq data has been deposited in GitHub and Zenodo repositories (https://doi.org/10.5281/zenodo.18781500 and https://doi.org/10.5281/zenodo.18781450). All information is included in the code availability section of the manuscript.

For manuscripts utilizing custom algorithms or software that are central to the research but not yet described in published literature, software must be made available to editors and reviewers. We strongly encourage code deposition in a community repository (e.g. GitHub). See the Nature Portfolio guidelines for submitting code & software for further information.

Data

Policy information about availability of data

All manuscripts must include a data availability statement. This statement should provide the following information, where applicable:

- Accession codes, unique identifiers, or web links for publicly available datasets
- A description of any restrictions on data availability
- For clinical datasets or third party data, please ensure that the statement adheres to our policy

A data availability statement is included in the manuscript. All sequencing data generated in this study have been deposited in the GEO database under the accession codes: GSE295577 (ChIP-seq), GSE295580 (RNA-seq), and GSE295581 (Tn-seq).

Research involving human participants, their data, or biological material

Policy information about studies with human participants or human data. See also policy information about sex, gender (identity/presentation), and sexual orientation and race, ethnicity and racism.

Reporting on sex and gender	NA
Reporting on race, ethnicity, or other socially relevant groupings	*Please specify the socially constructed or socially relevant categorization variable(s) used in your manuscript and explain why they were used. Please note that such variables should not be used as proxies for other socially constructed/relevant variables (for example, race or ethnicity should not be used as a proxy for socioeconomic status).*<br>*Provide clear definitions of the relevant terms used, how they were provided (by the participants/respondents, the researchers, or third parties), and the method(s) used to classify people into the different categories (e.g. self-report, census or administrative data, social media data, etc.)*<br>*Please provide details about how you controlled for confounding variables in your analyses.*
Population characteristics	NA
Recruitment	NA
Ethics oversight	NA

Note that full information on the approval of the study protocol must also be provided in the manuscript.

Field-specific reporting

Please select the one below that is the best fit for your research. If you are not sure, read the appropriate sections before making your selection.

☒ Life sciences ☐ Behavioural & social sciences ☐ Ecological, evolutionary & environmental sciences

For a reference copy of the document with all sections, see nature.com/documents/nr-reporting-summary-flat.pdf

Life sciences study design

All studies must disclose on these points even when the disclosure is negative.

Sample size	No statistical test was used to determine sample size. These were based on well-established protocols that provide reliable and representative data. All sequencing experimental datasets represent the population average of millions of bacteria. Microscopy quantification was carried out with n=400 cells were biological repeat to ensure that a large number of cells were analysed equally across biological replicates.
Data exclusions	No data were excluded from the analyses
Replication	All experiments were performed at least twice to ensure reproducibility, and similar results were obtained throughout.
Randomization	Not relevant to this study. All strains were selected randomly for inoculation from plate and grown under the same conditions. Observable differences are due to the difference in the genotype of each strain.
Blinding	Not relevant to this study. Observed differences are due to the genotype of the analysed strain.

Reporting for specific materials, systems and methods

We require information from authors about some types of materials, experimental systems and methods used in many studies. Here, indicate whether each material, system or method listed is relevant to your study. If you are not sure if a list item applies to your research, read the appropriate section before selecting a response.

Materials & experimental systems

n/a	Involved in the study
☐	☒ Antibodies
☒	☐ Eukaryotic cell lines
☒	☐ Palaeontology and archaeology
☒	☐ Animals and other organisms
☒	☐ Clinical data
☒	☐ Dual use research of concern
☒	☐ Plants

Methods

n/a	Involved in the study
☐	☒ ChIP-seq
☒	☐ Flow cytometry
☒	☐ MRI-based neuroimaging

Antibodies

Antibodies used

Monoclonal α-FLAG M2-Peroxidase HRP-conjugated antibody (Merck, 1:5000 dilution) cat number A8592. Polyclonal antibody against GtaL (1:1000 dilution of anti-serum, custom synthesis by Biosynth Laboratories, UK). Polyclonal antibody against GafY (3:1000 dilution of anti-serum, custom synthesis by Biosynth Laboratories, UK). Secondary antibody: HRP-conjugated goat anti-rabbit (Abcam, 1:10,000 dilution), cat number 6721.

Validation

The specificity of all antibodies used in this study was verified against lysates from deletion mutant strains. Both polyclonal antibodies have been used and reported in previous publications (Tran & Le, Nature Communications, 2024). Validation of the commercial anti-FLAG antibody was based on the technical data sheets from the manufacturer.

Plants

Seed stocks

Not relevant

Novel plant genotypes

Not relevant

Authentication

Not relevant

ChIP-seq

Data deposition

☒ Confirm that both raw and final processed data have been deposited in a public database such as GEO.

☒ Confirm that you have deposited or provided access to graph files (e.g. BED files) for the called peaks.

Data access links
May remain private before publication.

Reviewer token edszuogwttovdgf (for the GSE295577 ChIP-seq data: https://www.ncbi.nlm.nih.gov/geo/query/acc.cgi?acc=GSE295577)

Files in database submission

Raw fastq sequencing files and processed files (MACS2 callpeak data) are deposited in the GEO database.
ChIP-seq file list:
TLE11: delta cdxB xylX::Pxyl-cdxB negative control rep 1
TLE12: delta cdxB xylX::Pxyl-cdxB negative control rep 2
TLE13: delta cdxB xylX::Pxyl-cdxB-flag rep 1
TLE14: delta cdxB xylX::Pxyl-cdxB-flag rep 2
TLE15: delta rogA delta cdxB xylX::Pxyl-cdxB negative control rep 1
TLE16: delta rogA delta cdxB xylX::Pxyl-cdxBnegative control rep 2
TLE17: delta rogA delta cdxB xylX::Pxyl-cdxB-flag rep 1
TLE18: delta rogA delta cdxB xylX::Pxyl-cdxB-flag rep 2

Genome browser session
(e.g. UCSC)

Not applicable because there is no UCSC browser for the reference genome of the bacterium Caulobacter crescentus NA1000. However, all processed data have been uploaded to GEO and are available to the public and ChIP-seq profiles are show in the manuscript.

Methodology

Replicates

Two biological replicates were performed for ChIP-seq experiments. Peaks described in the manuscript were further validated by biochemical experiments (surface plasmon resonance)

Sequencing depth	Reads were short (50-100 bp) and paired-end with high FastQC scores. The mean total number of reads per sample was 5.9 million of which 4.8 million mapped uniquely
Antibodies	ANTI-FLAG M2 Affinity Gel (Merck, cat number A2220) https://www.sigmaaldrich.com/GB/en/product/sigma/a2220
Peak calling parameters	All analysis was done using the Galaxy platform (https://usegalaxy.eu/). Reads were mapped with Hisat2 and peaks were called using MACS2 callpeak (comparing test sample to negative control (non-flag-tagged gene) with a significance cut-off q-value of <0.01)
Data quality	On average 5.9 million reads were mapped per sample and quality of reads was checked with FastQC (Galaxy platform).<br>The minimum FDR (q-value) for peak detection used was <0.01. We report the number of peaks <2-fold enrichment. All peaks are listed in Table S2 and Table S4. All peaks described in the manuscript were inspected visually in both biological replicate experiments.
Software	All data processing was done using the Galaxy platform (https://usegalaxy.eu/). For analysis of ChIP-seq data, Illumina short reads were mapped back to the Caulobacter crescentus NA1000 genome with Hisat2. The coverage at each nucleotide position was computed using bedtools genome-cov. MACS2 callpeak was used to call peaks. ChIP-seq profiles were plotted using GraphPad Prism software.
